# Phytohormone Profiling and Transcriptional Regulation During Fruit Peel Development in Wax Apple

**DOI:** 10.3390/plants15213394

**Published:** 2026-11-02

**Authors:** Xueqin Zhang, Liying Ma, Lixia Lin, Ruiyun Lai, Jianzhong Lin, Zanhua Zhong, Haibo Ouyang

**Affiliations:** 1 Fujian Key Laboratory of Subtropical Plant Physiology and Biochemistry, Fujian Institute of Subtropical Botany, Xiamen 361006, China; 2 Department of Agriculture and Forestry, Hainan Tropical Ocean University, Sanya 572022, China

**Keywords:** wax apple, metabolomics, transcriptomics, phytohormone, peel development, peel color

## Abstract

Wax apple (*Syzygium samarangense*) is valued for its appealing color and delicious taste and has gained increasing popularity, along with significant economic value. Previous research largely focused on orchard cultivation practices, fruit development stage management, and postharvest treatments aimed at improving yield, quality, and market profitability. Study of the peel color of the wax apple has been scarce, although the substance accumulations and pigment changes in the fruit peel are unclear. In this study, the maximum variation of fruit biological traits and peel color occurred between S2 and S3. A total of 946 metabolites were identified, of which 17 key phytohormone-related metabolites may be related to the change of fruit peel color, encompassing jasmonates (JAs), cytokinins (CTKs), gibberellins (GAs), and zeatin ribosides (ZRs). Using RNA sequencing, 212 expressed genes relating to plant hormone signal transduction were identified. Among these, 18, 39, and 11 differentially expressed genes (DEGs) were identified in S2-vs-S1, S3-vs-S2, and S4-vs-S3, respectively. Critical genes within the hormone signaling cascades—including ARF, GID1B, ARR, JAR, NCED1, and TGA—exhibited strong correlations with peel color. Furthermore, fifteen distinct expressed gene modules and six distinct compound modules were identified through weighted gene coexpression network analysis, of which the blue and green modules display a robust correlation between numerous distinctly expressed genes and phytohormone metabolites. Collectively, our findings offer novel insights into the combinatorial regulation of wax apple fruit development and peel color, serving as a valuable resource for functional genomics and providing a theoretical basis for the genetic improvement and cultivation management of the wax apple.

## 1. Introduction

Wax apple (*Syzygium samarangense*), commonly known as wax jambu or java apple, is a non-climacteric, evergreen tropical fruit tree of the family Myrtaceae and is native to the Andaman and Nicobar Islands as well as the Malaysian Archipelago [1,2]. It was introduced to China in the 17th century, and is now widely cultivated in Taiwan, Hainan, Fujian, Guangdong, and Guangxi [3]. Recent studies demonstrated that extracts from its leaves, roots, and stems possessed notable pharmacological properties and had been traditionally used to treat coughs, dysentery, inflammation, etc. [4,5]. The fruit is rich in amino acids, proteins, flavonoids, and carbohydrates and exhibits cough-relieving, antibacterial, and immunomodulatory activities [3,6]. Owing to its considerable medicinal and nutritional value, the wax apple has garnered increasing attention from researchers worldwide [6,7,8].

Peel color is an important indicator of fruit maturity and a key determinant of appearance quality and commercial value. It is jointly determined by chlorophyll degradation and the accumulation of colored pigments, such as carotenoids and anthocyanins [9,10,11,12]. Among the numerous factors governing peel color formation, phytohormones play an indispensable role [13]. Peel coloration is regulated by both endogenous and exogenous hormones. Low concentrations of gibberellin delay chlorophyll degradation in the fruit peel, whereas high concentrations accelerate it [14]. Exogenous application of abscisic acid (ABA) and methyl jasmonate (MeJA) upregulated anthocyanin biosynthesis-related genes, including PAL, C4H, CHI, DFR, and ANS, thereby promoting anthocyanin biosynthesis and accumulation [15,16]. Similarly, exogenous ethylene accelerates carotenoid accumulation in the peel, while gibberellin delays it, both acting through HLH1 [17,18]. In summary, fruit color development is a very complex metabolic process; in addition to sugar and light, plant hormones are the important regulatory factors affecting pigment synthesis.

In recent years, omics technologies have been widely employed to investigate phytohormones, providing a powerful approach for elucidating the molecular mechanisms underlying fruit growth, development, and ripening [19,20]. Phytohormone changes during fruit development have been profiled in several species, including strawberry [19], banana [20], tomato [21], papaya [22], and kiwifruit [23]. However, studies on the peel coloration of wax apple using omics approaches remain scarce, and phytohormone profiles coupled with transcriptional regulation during peel coloring have not yet been reported.

Wax apple fruit ripens approximately 60 days after flowering, during which carotenoids and anthocyanins accumulate in the peel, giving rise to its attractive coloration that appeals to consumers. Although phytohormones are known to play critical roles in fruit development and ripening, the mechanisms by which they regulate peel coloration in wax apple remain unclear. To elucidate the effects and molecular mechanisms of phytohormones in peel coloration, we analyzed fruit size, single fruit fresh weight, peel color, phytohormone contents, differentially expressed genes (DEGs), and differentially accumulated metabolites (DAMs) at four fruit developmental stages. A total of 946 metabolites were identified, including 17 kinds of phytohormone-related metabolites. Expression genes numbering 212, relating to plant hormone signal transduction, were identified among 48,392 expressed genes, including several pivotal genes such as ARF, GID1B, ARR, JAR, NCED1, and TGA. This study explored the influence of phytohormones on wax apple peel development and coloration, providing valuable insights into the regulatory mechanisms of peel color formation and laying a foundation for future molecular research and breeding programs in wax apple.

## 2. Results

### 2.1. Characteristics of Fruit Development and Peel Coloring

**Figure 1:**
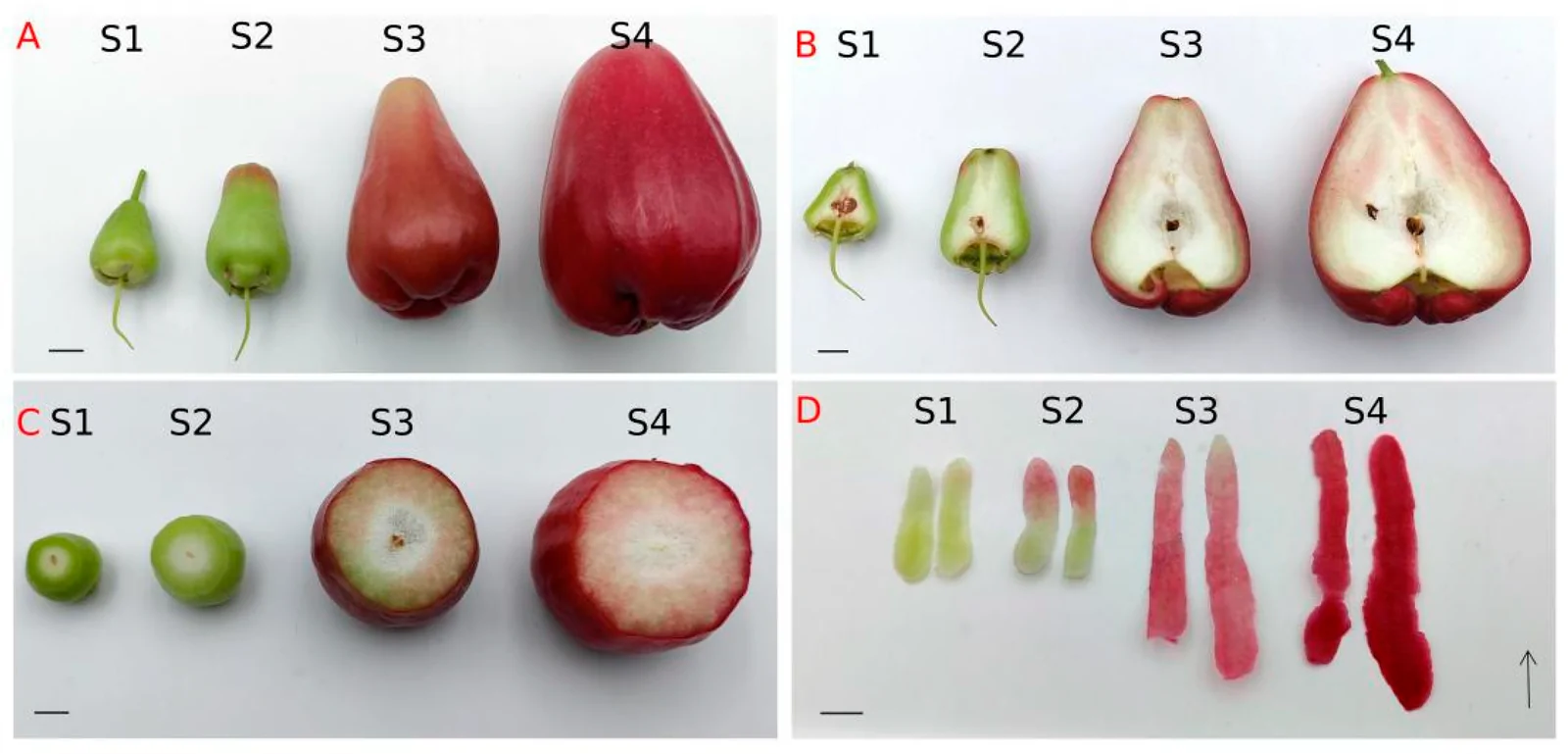
Four fruit growth and development stages in wax apple: (**A**) the whole fruit, (**B**) vertical section, (**C**) cross section, (**D**) peels of fruit by cutting from top to tip. Four stages: (S1) immature green stage (15 DAF), (S2) transition to ripening (30 DAF), (S3) light red stage (45 DAF), (S4) full red ripened stage (60 DAF). “—” in the picture represents one centimeter.

The fruit of the wax apple is narrow at the tip and wide at the base, bearing four fleshy calyx lobes. Its peel is smooth and covered with a layer of wax, giving the fruit an attractive appearance. Under natural conditions, the wax apple blossoms in March and April, and the fruit ripens approximately 60 days after flowering (DAF). Four fruit developmental stages are shown in Figure 1: the immature green stage (S1, 15 DAF), the transition stage (S2, 30 DAF), the light red stage (S3, 45 DAF), and the full red ripe stage (S4, 60 DAF). Whole fruits, vertical and cross sections, and peels from the distal to proximal end relative to the stalk are presented in Figure 1. The flesh color changes from green to pale red and finally to red from S1 to S4, and the fruit contains white spongy tissue in the center, as revealed by vertical and cross sectioning. The core of the fruit is hollow, and its cavity expands as the fruit ripens.

**Figure 2:**
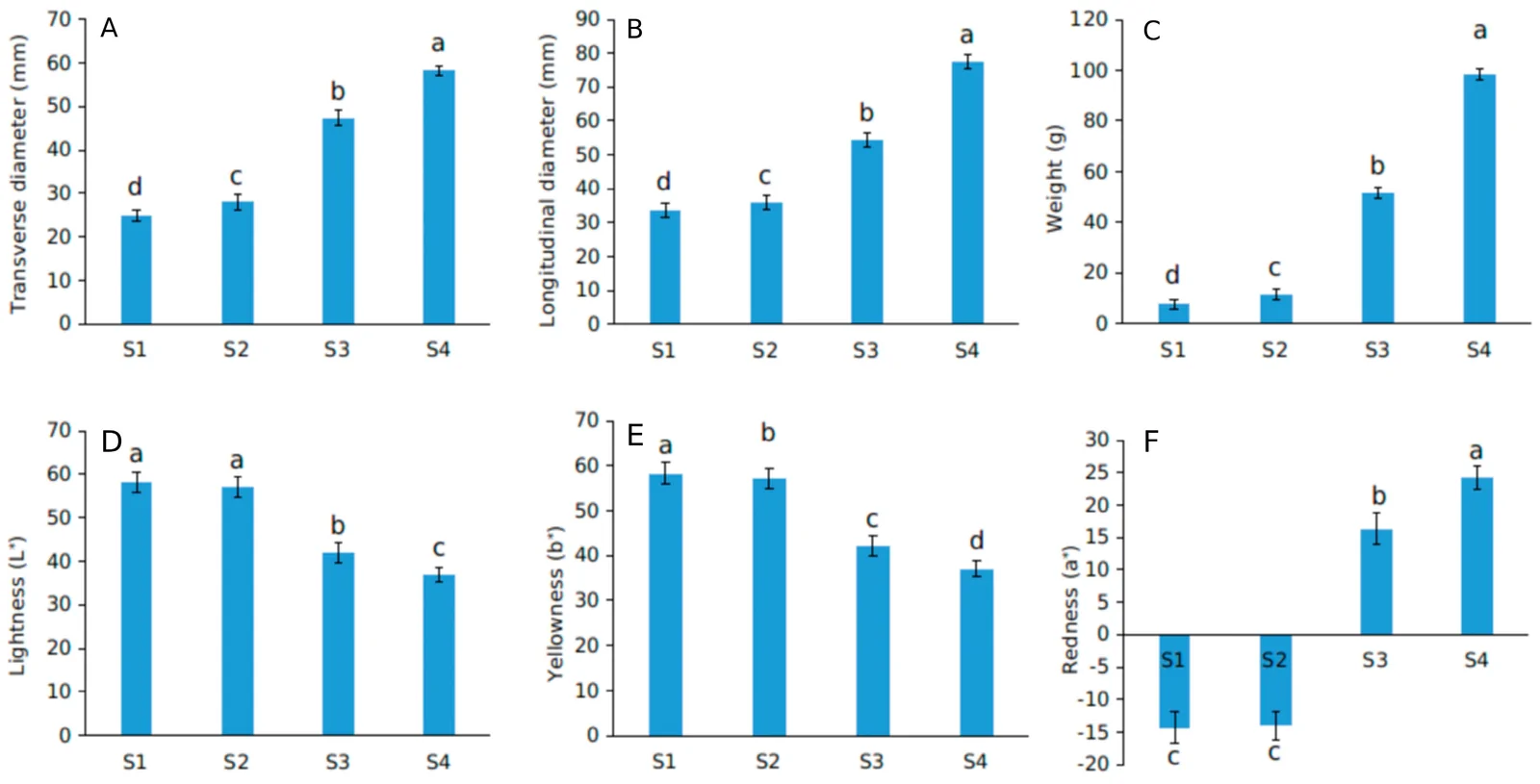
Biological characteristics of fruit and peel color: (**A**) transverse diameter, (**B**) longitudinal diameter, (**C**) weight of single fruit, (**D**) lightness of peel, (**E**) yellowness of peel, (**F**) redness of peel. Different letters above or below the bars indicate significant differences by Duncan’s test (*p* < 0.05).

Fruit size and weight is pivotal to fruit tree production, directly influencing fruit quality and playing a crucial role in determining yield and commercial value. Phenotypic analysis showed that the transverse diameter of the fruit increased from 24.97 to 58.29 mm, while the longitudinal diameter increased from 33.71 to 77.59 mm. Likewise, single fruit weight gradually increased from 7.62 to 98.43 g (Figure 2A–C). The smallest variation in these fruit traits occurred between S1 and S2, followed by that between S3 and S4, whereas the greatest variation occurred from S2 to S3. Notably, the transverse diameter, longitudinal diameter, and single fruit weight at S3 were 1.90-, 1.62-, and 6.78-fold higher than those at S2, respectively.

Fruit color is a significant indicator of fruit quality. The results showed that lightness (L*) and yellowness (b*) decreased while redness (a*) increased as the fruit developed. L* declined significantly from 58.23 to 36.98, and b* decreased from 30.49 to 12.13, while a* increased from −14.31 to 24.17 (Figure 2D–F). There were no significant differences in L* and a* between S1 and S2, but both differed significantly between S1/S2 and S3/S4. In contrast, b* showed significant difference between different stages. 

### 2.2. Metabolites Related to Phytohormones

Phytohormones play a crucial role in regulating fruit development, ripening, and quality formation, as was demonstrated in previous studies. Elucidating the interactions among different phytohormones and the signal transduction networks they form is essential to understand the regulation of fruit development and ripening. Wax apple fruit is rich in primary and secondary metabolites, such as amino acids and their derivatives, flavonoids, sugars and their derivatives, and phenolic compounds, which collectively determine fruit quality, flavor, and peel coloration. A total of 946 metabolites were detected across the four developmental stages, including amino acids and derivatives, flavonoids, carbohydrates and derivatives, lipids, and others [24].

Among the 946 detected metabolites, 17 were phytohormone-related (Table 1), belonging to eight classes: jasmonates (JAs), cytokinins (CTKs), gibberellins (GAs), zeatin ribosides (ZRs), indoleacetic acid–glutamic acid (IAA), abscisic acid (ABA), 1-aminocyclopropane carboxylic acid (ACC), and salicylic acid (SA). 1-aminocyclopropane carboxylic acid, 9-β-glucosyl-N6-(isoprene) adenine, isopentenyl adenosine, gibberellin A7, and trans-zeatin 9-O-glucoside were down-regulated in S2-vs-S1 but up-regulated in S3-vs-S2 and S4-vs-S3, whereas abscisic acid showed the opposite pattern. Indoleacetic acid–glutamic acid, salicylic acid O-glucoside, and gibberellin A20 were up-regulated in S3-vs-S2 but down-regulated in S2-vs-S1 and S4-vs-S3. Gibberellin A9 and N-[(-)-jasmonoyl]-(L)-isoleucine were up-regulated in S4-vs-S3 but down-regulated in S2-vs-S1 and S3-vs-S2. Isoprenyl-7-glucoside was up-regulated in S2-vs-S1 and S4-vs-S3 but down-regulated in S3-vs-S2, whereas trans-zeatin N-glucoside showed the opposite pattern. In contrast, jasmonic acid, 12-hydroxyjasmine monoacyl-L-isoleucine, trans-Zeatin riboside, and N-[(-)-jasmonoyl]-(L)-isoleucine decreased progressively during fruit development.

### 2.3. Differential Gene Expression During Peel Development

To further elucidate the potential molecular regulatory mechanisms underlying phytohormone-mediated peel color during fruit development and ripening, transcriptome sequencing was performed on wax apple peels. A total of 81.84 Gb raw bases and 80.98 Gb clean bases were generated. The average clean base yields for the four stages (S1–S4) were 6.94, 6.92, 6.10, and 7.32 Gb, respectively. The valid base rate ranged from 98.76% to 99.14%, and the GC content ranged from 49.43% to 50.20%. The Q20 and Q30 scores were above 97.24% and 92.70%, respectively (Supplementary Table S1), indicating that the sequencing data was of high quality and suitable for subsequent analysis.

**Figure 3:**
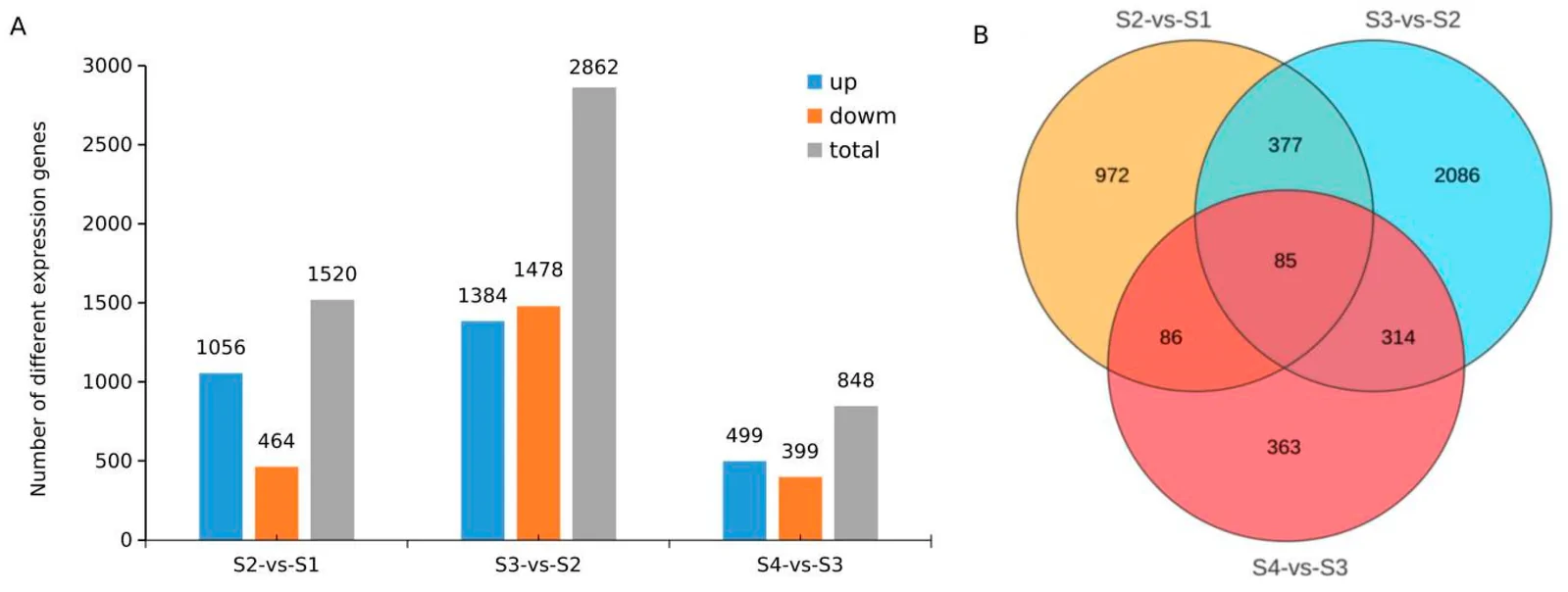
Differentially expressed genes (DEGs) in peels: (**A**) numbers of DEGs in different comparision; (**B**) Venn diagram of DEGs.

Pairwise comparisons of the peel transcriptomes across the four developmental stages identified 1520, 2862, and 848 DEGs in S2-vs-S1, S3-vs-S2, and S4-vs-S3, respectively (Figure 3A, Supplementary Table S2). Among these, 85 DEGs were shared by all three comparison groups, while 462, 171, and 399 DEGs were shared between S2-vs-S1 and S3-vs-S2, S2-vs-S1 and S4-vs-S3, S3-vs-S2 and S4-vs-S3 (Figure 3B). KEGG enrichment analysis revealed 107, 117, and 81 significantly enriched pathways in S2-vs-S1, S3-vs-S2, and S4-vs-S3, respectively. Notably, a total of 212 genes were obtained relating to plant hormone signal transduction among 48,392 expressed genes, including 18, 39, and 11 DEGs in S2-vs-S1, S3-vs-S2, and S4-vs-S3, respectively.

### 2.4. KEGG Pathway Analysis of DEGs

**Figure 4:**
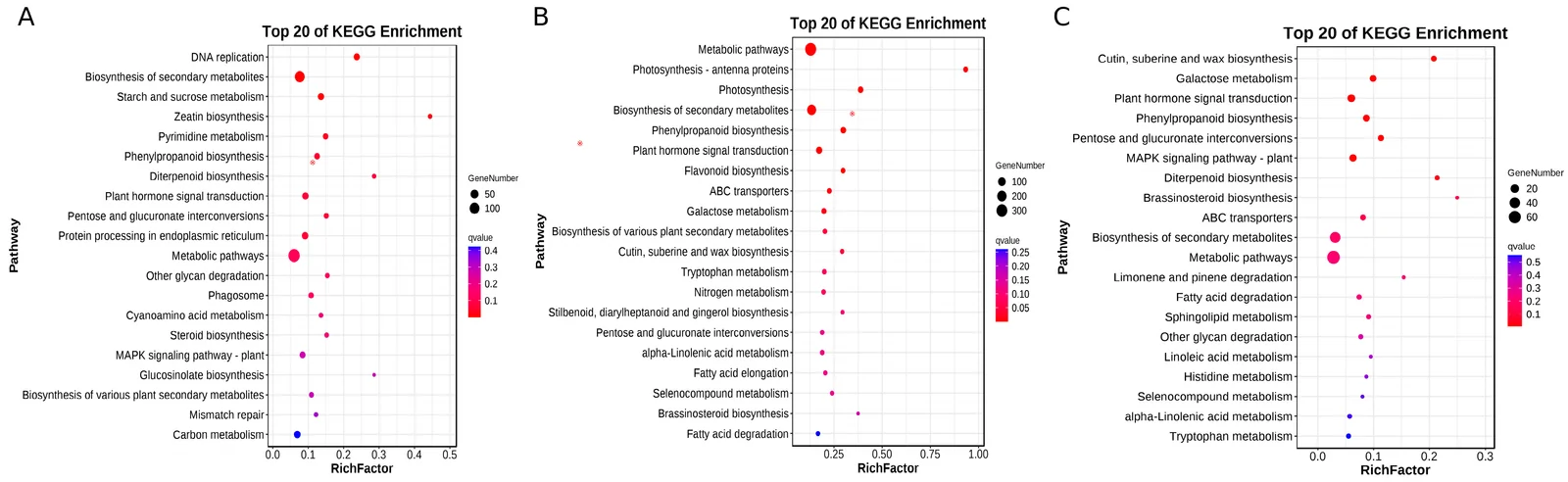
KEGG enrichment pathway of DEGs in peels: (**A**) KEGG enrichment pathway in S2-vs-S1; (**B**) KEGG enrichment pathway in S3-vs-S2; (**C**) KEGG enrichment pathway in S4-vs-S3.

To functionally characterize the DEGs, KEGG classification and annotation were performed to identify the major pathways involved. The DEGs were assigned to five KEGG pathway categories: metabolism; genetic information processing; environmental information processing; cellular processes; and organismal systems. A total of 124 pathways were enriched, and the top 20 significantly enriched pathways in each comparison group are shown in Figure 4A–C. Among these, pathways related to peel development included five phytohormone-associated pathways: biosynthesis of secondary metabolites (ko01110), plant hormone signal transduction (ko04075), flavonoid biosynthesis (ko00941), tryptophan metabolism (ko00380), and brassinosteroid biosynthesis (ko00905). Notably, plant hormone signal transduction was among the most significantly enriched pathways, ranking 8th, 6th, and 3rd in S2-vs-S1 (Figure 4A), S3-vs-S2 (Figure 4B), and S4-vs-S3 (Figure 4C), respectively.

### 2.5. Phytohormone Signal Transduction Pathways in Peel Development

Fruit coloration is a complex process regulated by multiple factors, among which phytohormones play a critical regulatory role. Phytohormones are of great significance in fruit color formation, with their contents dynamically increasing or decreasing during fruit development and ripening. Peel coloration is not controlled by a single hormone acting at a specific stage; rather, it results from a complex network in which multiple hormones jointly regulate various aspects of fruit development and ripening.

In recent years, hormonal regulation of fruit development and ripening has been investigated increasingly in strawberry, papaya, and litchi. These studies demonstrated that auxin (IAA), cytokinins (CTKs), gibberellins (GAs), abscisic acid (ABA), brassinosteroids (BRs), ethylene (ETH), and jasmonic acid (JA) play critical roles in fruit development and ripening. However, information on phytohormones in wax apple remains limited.

#### 2.5.1. Auxin (IAA)

**Figure 5:**
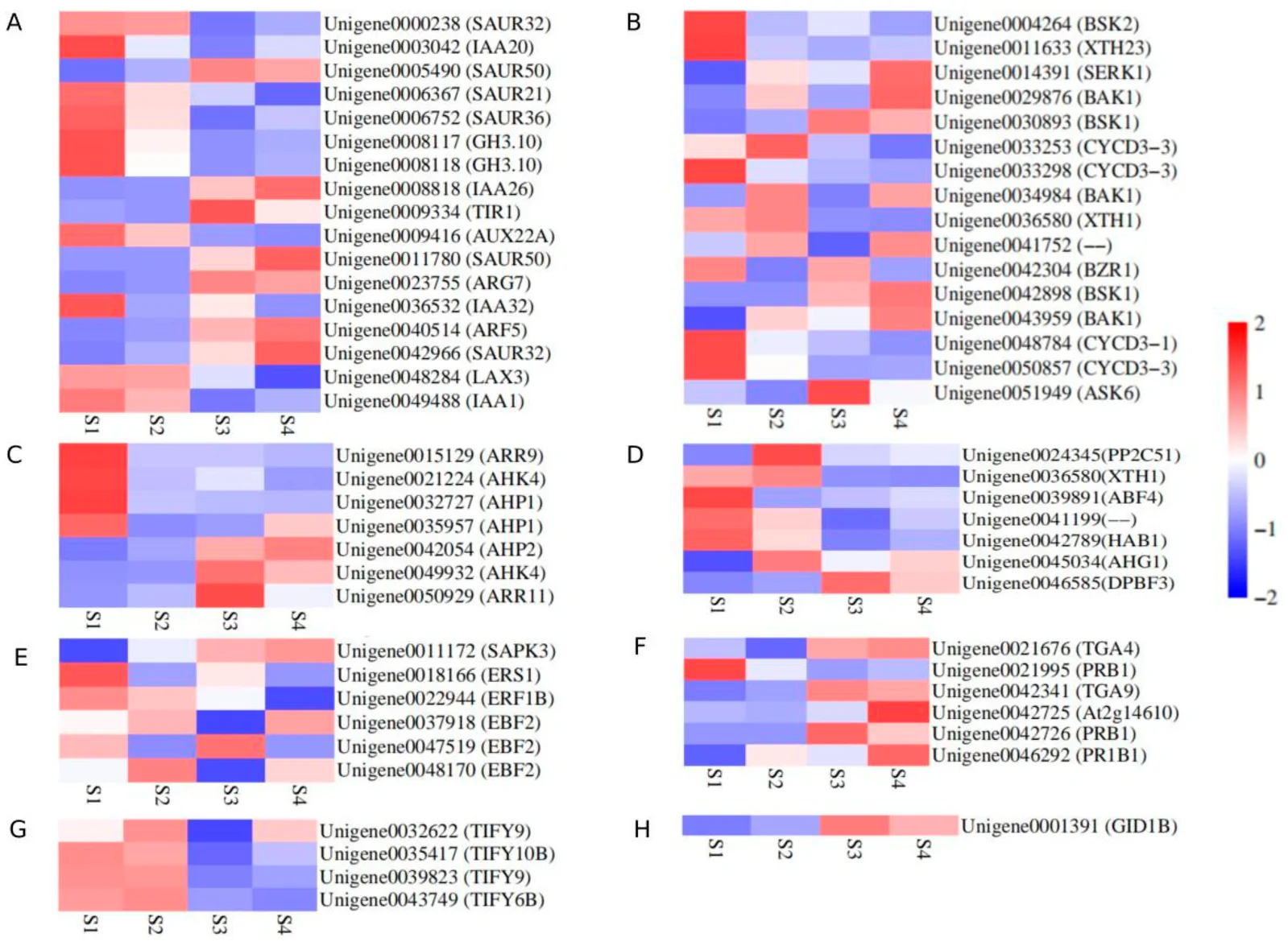
Phytohormone-related gene expression level in peel of wax apple: (**A**) auxin; (**B**) brassinosteroid; (**C**) cytokinine; (**D**) absicisic acid; (**E**) ethylene; (**F**) salicylic acid; (**G**) jasmonic acid; (**H**) gibberllin.

IAA promotes cell division and fruit enlargement, and its endogenous content decreases during fruit coloration and ripening. IAA negatively regulates peel coloration by inhibiting anthocyanin synthesis and delaying chlorophyll degradation through the AUX/IAA–ARF pathway. It also interacts with ethylene and ABA to coordinate peel color development. In this study, 17 DEGs were annotated to the IAA signal transduction pathway (Figure 5A), including seven small auxin-up RNA (SAUR) genes encoding auxin-responsive factors, four auxin/indole-3-acetic acid (AUX/IAA) repressor genes, two auxin-responsive GH3 genes, one AUXIN/AAUX receptor gene (AUX1), one auxin response factor (ARF) gene, one auxin influx carrier (LAX) gene, and one transport inhibitor response (TIR1) gene. Notably, all TIR1 and ARF genes were up-regulated, GH3 and AUX/IAA genes were down-regulated, whereas SAUR genes were either up- or down-regulated.

#### 2.5.2. Brassinosteroid (BR)

The content of endogenous BR increases during fruit coloration and ripening. BR positively regulates peel coloration and acts synergistically with ethylene and ABA to promote fruit coloration. In this study, 16 DEGs were annotated to the BR signal transduction pathway (Figure 5B), including five brassinosteroid insensitive 1-associated receptor kinase 1 genes (SERK1, unigene0041752, and three unigenes annotated as BAK1), three BR-signaling kinase genes (BSK, BSK1, and SK2), one brassinosteroid resistant 1/2 gene (BZR1), one protein brassinosteroid insensitive 2 gene (ASK6), two xyloglucan endotransglucosylase/hydrolase genes (XTH1 and XTH23), and four cyclin D3 genes (CYCD3-1 and CYCD3-3). Among them, BAKs and CYCD3s were up-regulated, whereas BSKs were down-regulated.

#### 2.5.3. Cytokinin (CTK)

The action and the change pattern of CTK were similar to those of IAA during fruit development and ripening. High concentrations of CTK inhibit peel coloration by suppressing anthocyanin synthesis through B-type ARRs and by antagonizing the ABA-mediated coloration pathway. In this study, seven DEGs were annotated to the cytokinin signal transduction pathway (Figure 5C), including three genes encoding histidine-containing phosphotransfer proteins (AHP1, AHP2, and AHP2), two response regulator genes (ARR9 and ARR11), and two cytokinin receptor histidine kinase genes (AHK4 and AHK4). ARR9 was down-regulated, whereas ARR11 was up-regulated at the early stages and down-regulated at S4. AHP and AHK genes were either up- or down-regulated.

#### 2.5.4. Abscisic Acid (ABA)

Abscisic acid (ABA) positively regulates fruit coloration by activating the MBW (MYB–bHLH–WD40) transcriptional complex through the SnRK2–ABF signaling pathway, thereby promoting anthocyanin synthesis and pigment accumulation. ABA also forms a complex hormonal regulatory network with IAA, CTKs, BRs, and ETH to coordinate peel color changes. In this study, seven DEGs were annotated to the ABA signal transduction pathway (Figure 5D), including four encoding protein phosphatase 2Cs (PP2C51, unigene0041199, HAB1, and AHG1), two ABA-responsive element binding factors (ABF4 and DPBF3), and one xyloglucan endotransglucosylase/hydrolase gene TCH4(XTH1). Among them, XTH1, HAB1, ABF4, and unigene0041199 were down-regulated, whereas DPBF3, AHG1, and PP2C51 were up-regulated at the early stages and down-regulated at the later stages.

#### 2.5.5. Other Signal Transduction Pathways

**Figure 6:**
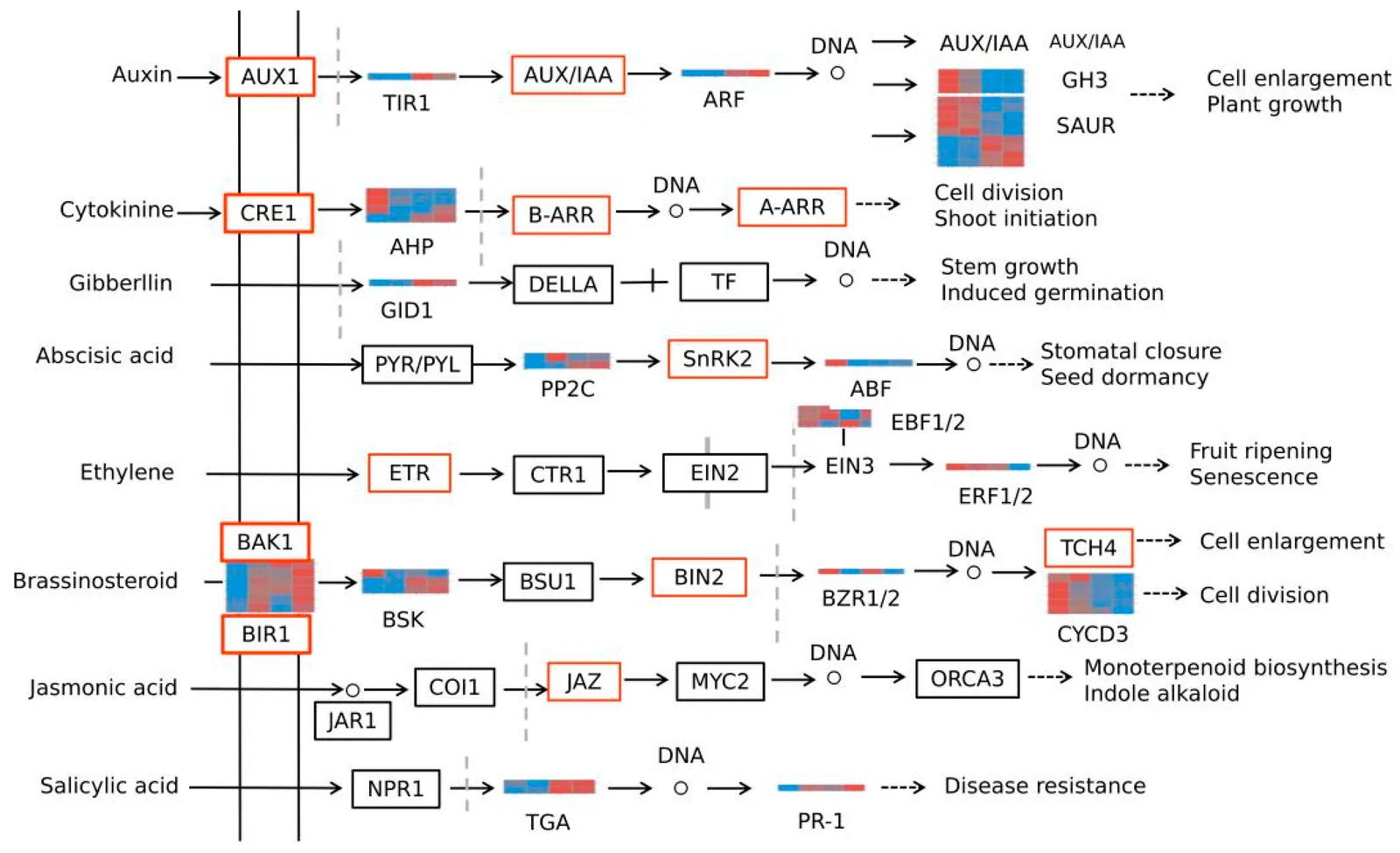
Plant hormone signal transduction in peel of wax apple. Heat map representing the expression of genes associated with plant hormone signal transduction at four different development stages. Up-regulated genes marked in red box, down-regulated genes marked in blue box.

In addition to IAA, BRs, CTKs, and ABA, other plant hormones also participate in the growth, development, and peel coloration of wax apple fruit (Figure 5E–H and Figure 6). Six DEGs were annotated to the ethylene signal transduction pathway (Figure 5E), including three EIN3-binding F-box protein genes (EBF2, EBF2, and EBF2), one ethylene receptor gene (ERS1), one ethylene-responsive transcription factor gene (ERF1B), and one serine/threonine protein kinase gene (SAPK3). ERF1B was down-regulated, whereas SAPK3 was up-regulated. EBF2 (Unigene0037918 and Unigene0048170) was up-regulated at the early stages and down-regulated at the later stages, whereas EBF2 (Unigene0047519) showed a down–up–down expression pattern. Six DEGs were annotated to the salicylic acid signal transduction pathway (Figure 5F), including two TGA transcription factors (TGA4 and TGA9) and four pathogenesis-related protein genes (PRB1, PRB1, PR1B1, and unigene0042725). Among them, PRB1 (Unigene0021995) was down-regulated, whereas all other genes were up-regulated. Four jasmonate ZIM domain (JAZ)-containing protein genes (TIFY6B, TIFY9, TIFY9, and TIFY10B), associated with the jasmonic acid signal transduction pathway, were all down-regulated (Figure 5G). Only one DEG was annotated to the GA signal transduction pathway (Figure 5H)—the gibberellin receptor GID1 (GID1B)—which was up-regulated during fruit growth and development.

### 2.6. Dynamic Expression of Key Regulatory Genes During Peel Development

Phytohormones, such as auxin (IAA), gibberellins (GAs), and cytokinins (CTKs), play critical regulatory roles during fruit growth, ripening, and peel coloration [18,25]. Auxin directly regulates these processes through Aux/IAA proteins and auxin response factors (ARFs), whereas GAs bind to their receptor GID1, which in turn interacts with DELLA proteins, the negative regulators of GA responses. Cytokinins promote cell division and delay fruit coloration via response regulator genes (ARRs), while abscisic acid (ABA) regulates fruit development and ripening through 9-cis-epoxycarotenoid dioxygenase (NCED), a key enzyme in ABA biosynthesis. Among the critical signaling components, jasmonate ZIM-domain (JAZ) proteins are key regulators ubiquitously present in jasmonate signaling, and TGA transcription factors, members of the basic region leucine zipper (bZIP) family, are involved in salicylic acid synthesis and signal transduction during fruit growth and development.

**Figure 7:**
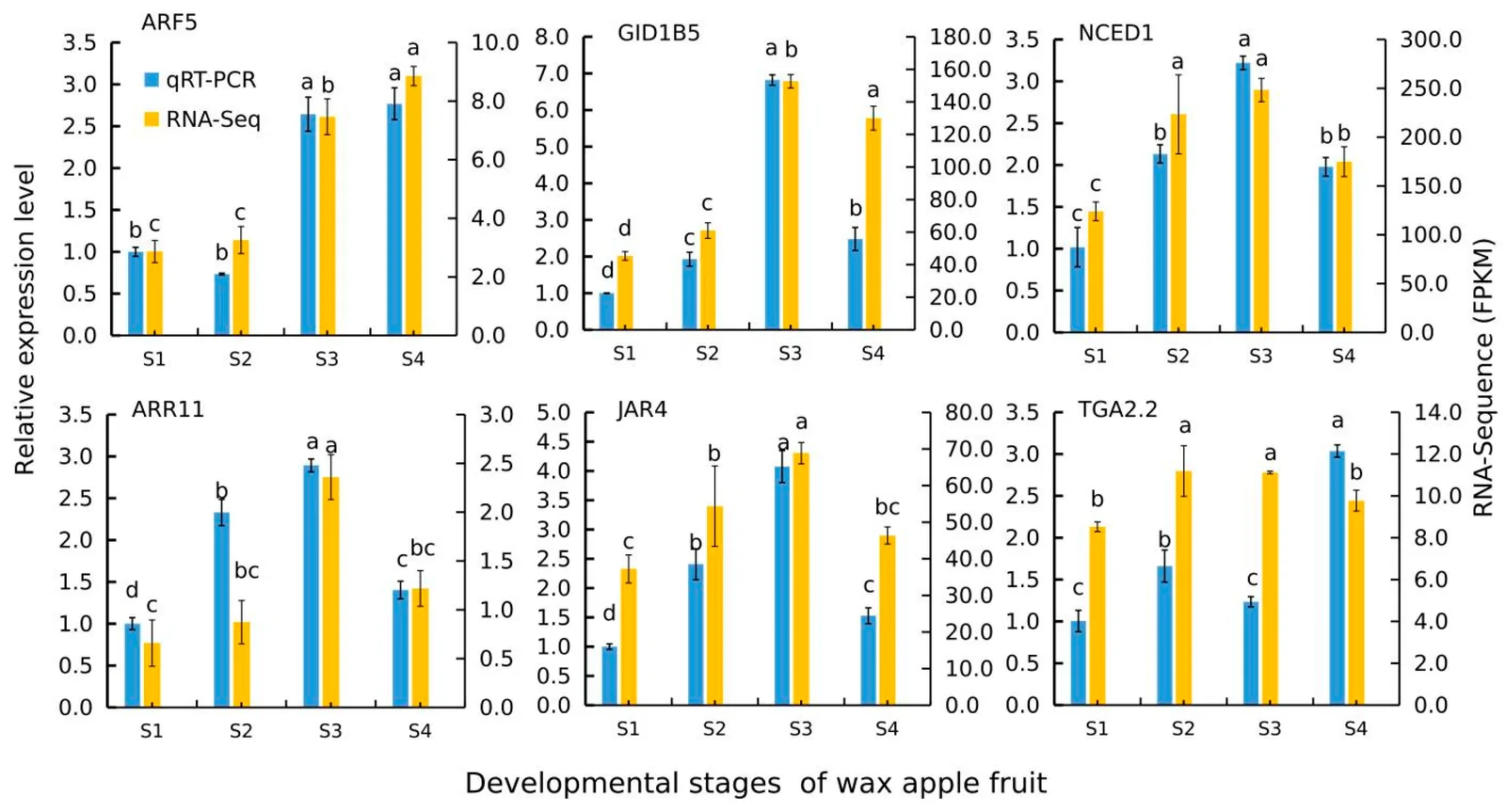
RT–PCR validation of the expression profiles of six phytohormone-related genes. Different letters above or below the bars indicate significant differences within a gene (*p* < 0.05) by Duncan’s test.

To validate the transcriptome data, six genes involved in plant hormone signal transduction pathways were selected for reverse transcription real-time quantitative PCR (RT–qPCR) analysis. The expression trends of all six genes were consistent with the RNA-seq results, confirming the reliability of the transcriptome data (Figure 7). The FPKM value of ARF5 increased gradually and peaked at S4. The FPKM values of NCED1, JAR4, GID1B, and ARR11 increased gradually, peaked at S3, and then decreased. The FPKM values of TGA2.2 increased and then decreased. Consistent with the RNA-seq results, RT–qPCR analysis showed that the relative expression level of ARF5 decreased first and then increased gradually; its lowest level was at S2 and its peak at S4. The relative expression level of NCED1, JAR4, GID1B, and ARR11 increased gradually, peaked at S3, and then decreased, whereas the relative expression level of TGA2.2 fluctuated across the stages—increasing, decreasing, and increasing again—with its peak at S4.

### 2.7. WGCNA Analysis of the Metabolic Compounds

WGCNA was performed on the transcriptomic and metabolomic data from peel samples at four developmental stages (12 samples in total), encompassing 48,392 expressed genes and 946 metabolite compounds. Fifteen gene coexpression modules (containing 17,254 genes) and six metabolite modules (containing 808 compounds) were identified using the dynamic tree cutting method. Among these, 155 genes and 17 compounds were annotated as phytohormone-related (Supplementary Table S3).

**Figure 8:**
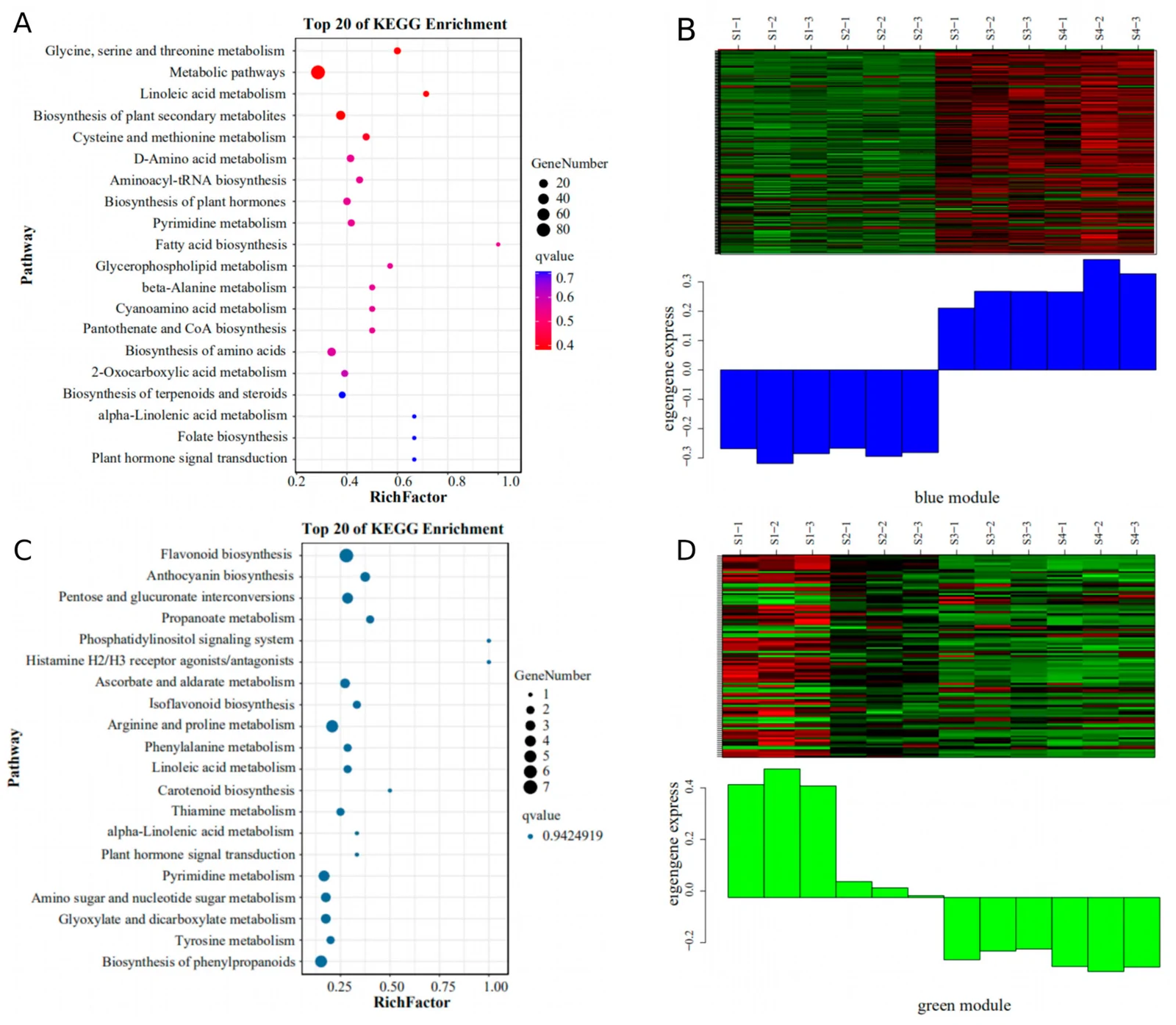
Weighted gene coexpression network analysis: (**A**) KEGG enrichment analysis in MM.blue, (**B**) expression trends of DAMs in MM.blue, (**C**) KEGG enrichment analysis in MM.green, (**D**) the expression trend of DAMs in MM.green.

Correlation analysis was performed between the fifteen gene coexpression modules and the six metabolite modules. The results showed that hormone-related genes were enriched in the plant hormone signal transduction pathway in MM.blue (Figure 8A) and MM.green (Figure 8C), whereas hormone-related metabolites were enriched in the plant hormone biosynthesis pathway in MM.blue (Figure 8B) and MM.green (Figure 8D). Specifically, MM.blue contained 35 hormone-related genes and four plant hormone-related metabolites, while MM.green contained 29 hormone-related genes and three plant hormone-related metabolites (Supplementary Table S3).

### 2.8. Phytohormone Dynamics During Peel Development

**Figure 9:**
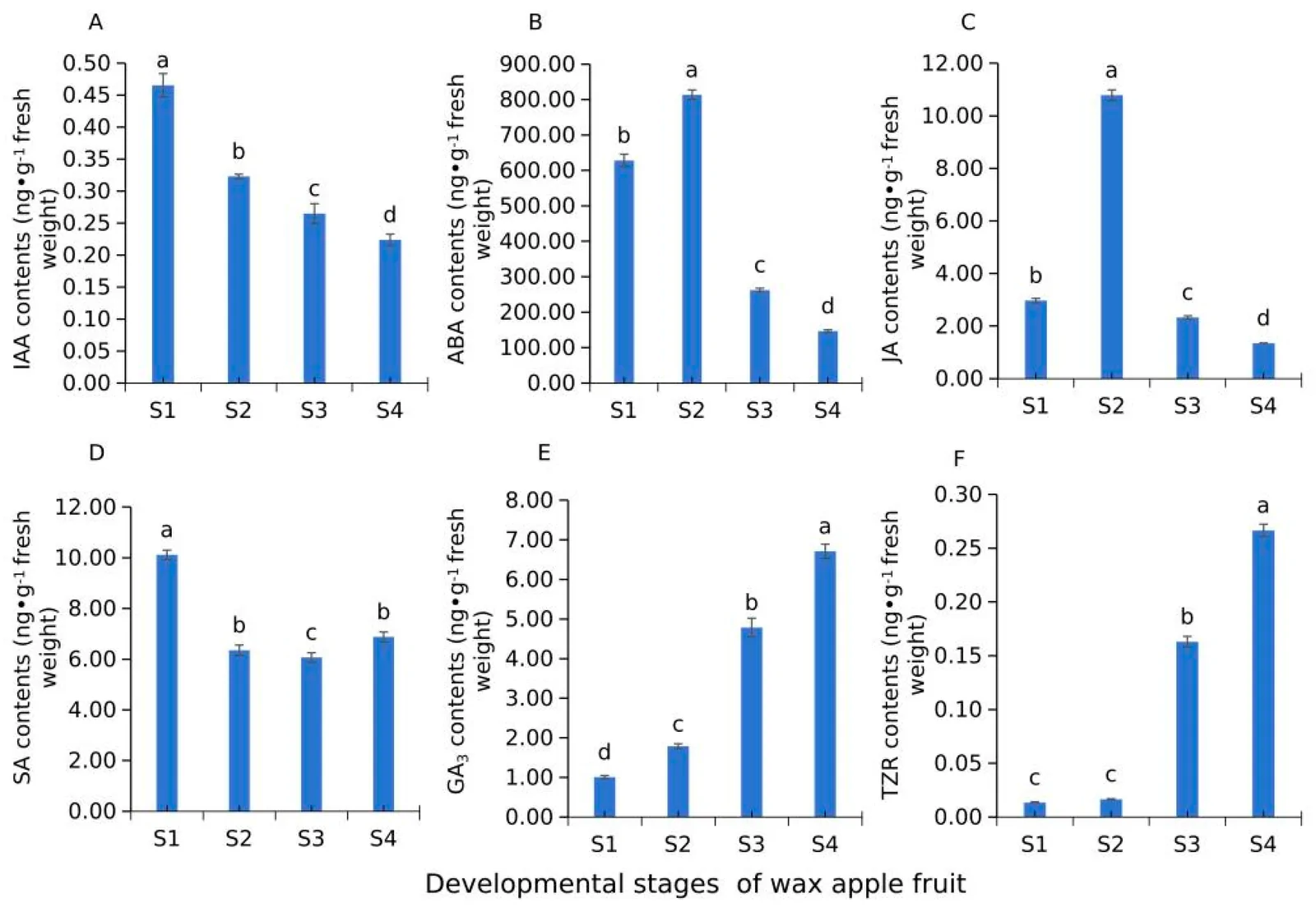
Phytohormone content in peels of wax apple at different developmental stages of fruit. (**A**) IAA content, (**B**) ABA content, (**C**) JA content, (**D**) SA content, (**E**) GA_3_ content, (**F**) TZR content. Different letters above or below the bars indicate significant differences by Duncan’s test (*p* < 0.05).

The results showed that IAA content decreased gradually during development: the IAA content at S1 was 1.44-, 1.76-, and 2.08-fold higher than that at S2, S3, and S4, respectively (Figure 9A). In contrast, ABA and JA contents first increased and then decreased, peaking at S2 (Figure 9B,C). The ABA content at S2 was 1.29-, 3.01-, and 5.55-fold, while the JA content was 3.63-, 4.63-, and 7.98-fold higher than that at S1, S3, and S4, respectively. SA content first decreased and then increased, reaching its maximum at S1 and minimum at S3 (Figure 9D); the SA content at S1 was 1.59-, 1.67-, and 1.47-fold higher than that at S2, S3, and S4, respectively. GA_3_ and TZR contents increased gradually, both reaching their maximum at S4 (Figure 9E,F). The GA3 content at S4 was 6.68-, 3.75-, and 1.40-fold, while the TZR content was 19.73-, 16.12-, and 1.63-fold higher than that at S1, S2, and S3, respectively. Statistical analysis revealed significant differences in IAA, ABA, JA, and GA_3_ contents among the four stages. The SA content did not differ significantly between S2 and S4 but differed significantly from that at S1 and S3. Similarly, the TZR content showed no significant difference between S1 and S2 but differed significantly from that at S3 and S4.

### 2.9. A Presumptive Model of Peel Color Regulated by Phytohormones

**Figure 10:**
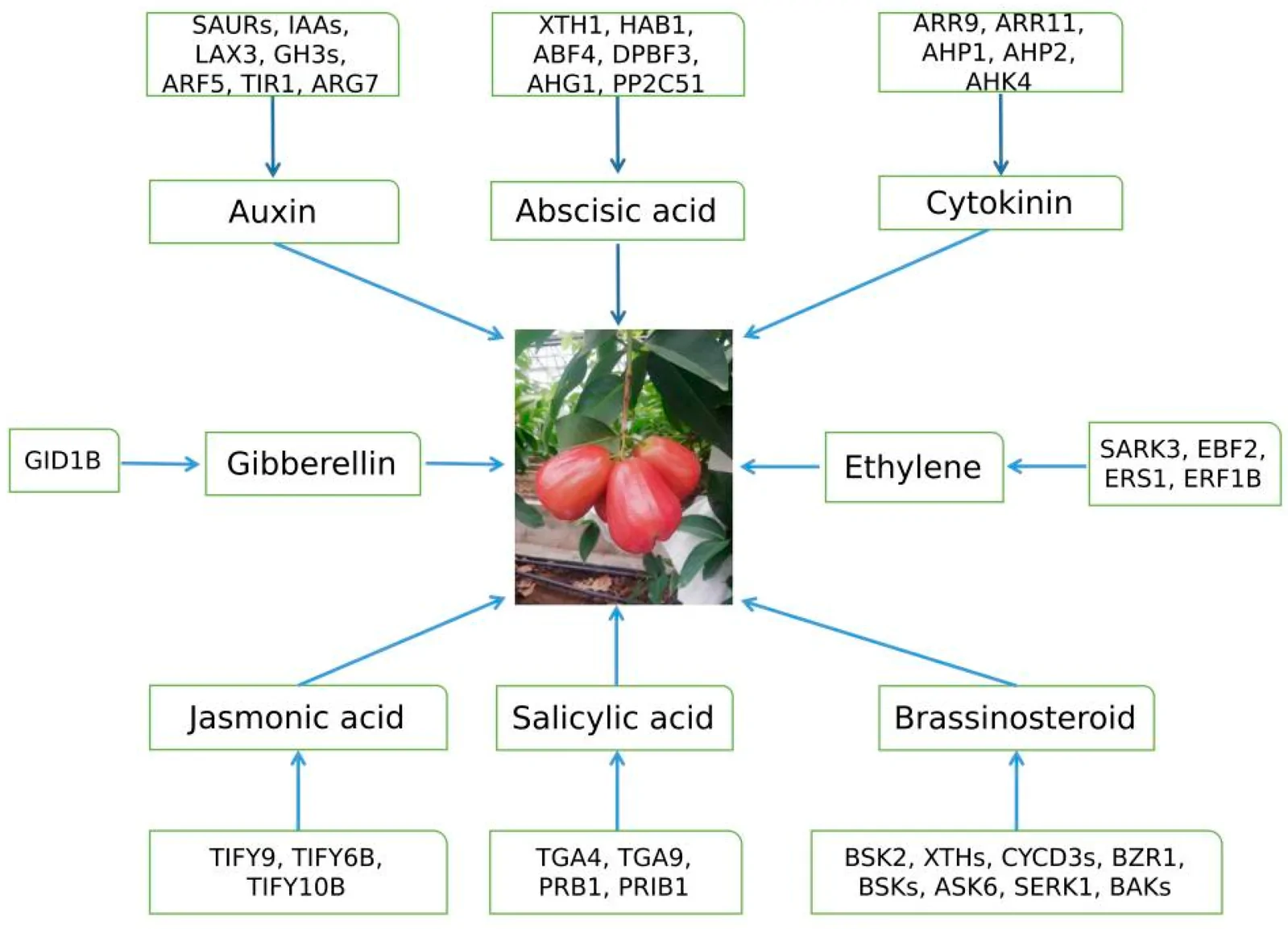
A presumptive model of peel color regulated by phytohormones in wax apple.

Based on the biological characteristics of the fruit, the changes in peel color, and the metabolomic and transcriptomic analyses of phytohormones in peels at different fruit growth and developmental stages, a presumptive model for the regulation of peel color by phytohormones in wax apple was proposed (Figure 10). In this model, phytohormones may play an important role in controlling and regulating peel coloration. This study provides valuable insights for the application of plant growth regulators to manipulate peel coloring in wax apple and serves as a reference for subsequent research and further molecular functional analyses. These will facilitate the breeding of superior wax apple varieties and accelerate molecular breeding.

## 3. Discussion

### 3.1. Phytohormones Play Important Roles in Regulating Peel Coloring

The contents and levels of phytohormones, such as IAA, JA, GA, SA, and ABA, change constantly during fruit development and ripening [11,26]. During the fruit development of wax apple, the period from S1 to S2 was characterized by slow growth, S2 to S3 represented a critical stage of fruit expansion, and S3 to S4 was the critical stage of fruit ripening and quality formation. Accordingly, peel color changed gradually from green at S1, through green and pale red at S2 and S3, to red at S4, accompanied by substantial changes in lightness (L*), redness (a*), and yellowness (b*): L* and b* decreased while a^*^ increased as the fruit developed. Phytohormones play important roles in regulating peel coloration, and the contents and expression levels of related genes differed significantly among the comparison. Specifically, IAA content decreased, whereas the contents of GA_3_ (a kind of gibberellin) and TZR (a kind of cytokinin) increased gradually; ABA and JA contents first increased and then decreased, while SA content showed the opposite trend during peel coloration. These dynamic changes in phytohormones in wax apple peels are similar to those observed in tomato [21], mango [27], strawberry [28,29], and banana [30].

### 3.2. Phytohormone-Related Genes Regulate the Formation of Peel Color

In this study, the number of DEGs in S3-vs-S2 was the highest, suggesting that this stage involved the most complex physiological and biochemical changes. The period from S3 to S4 was critical for fruit ripening, during which significant changes in color, sugar content, and volatile compounds occurred—consistent with findings in strawberry [19,27,28], citrus [31,32], onion [33], pineapple [34], and loquat [35,36]. Phytohormone-related genes play important roles in regulating peel coloration. The expression of IAA, CTK, SA, and ABA pathway genes was either up- or down-regulated, whereas GA pathway genes were predominantly up-regulated and JA pathway genes down-regulated, which is similar to the patterns reported in papaya [22], strawberry [19,28,29], citrus [31,32], and loquat [35,36]. During peel coloration of wax apple, numerous phytohormone-related genes played critical roles, such as TIR1 and ARF in the auxin pathway, AHP in the cytokinin pathway, and TGA and PR-1 in the salicylic acid pathway (Figure 6 and Figure 10). Jasmonoyl-L-isoleucine and abscisic acid are involved in the growth, development, and ripening of wax apple fruit, although their specific functions remain to be verified. The specific functions, dynamic changes, and regulatory mechanisms of genes involved in plant hormone signal transduction pathways warrant further investigation.

The results showed that although phytohormones and phytohormone-related genes played important roles in regulating peel coloration, differentially accumulated phytohormone-related metabolites (DAMs) were scarce during this process. Plant hormone contents in fruits are inherently low, and their accurate quantification is highly dependent on the sensitivity of detection methods and instruments. This suggests that phytohormones mainly affect fruit size, peel color, ripening, and post-harvest preservation, while having limited effects on phytohormone-related metabolite accumulation. This may be regulated primarily at the transcriptional level—through receptors and signal transduction pathways—with relatively minor effects on the accumulation of downstream metabolites. Similar results have been reported in kiwifruit [23], litchi [25], and strawberry [28,29]. Many studies have integrated transcriptomic and metabolomic analyses to reveal the mechanisms underlying flavonoid [37,38] and volatile compound accumulation in fruits [39], but fewer studies have focused on phytohormone-related DAMs, possibly due to the limited number of such metabolites [40]. In this study, weighted gene coexpression network analysis (WGCNA) revealed that the blue and green modules were strongly correlated with numerous expressed genes and phytohormone metabolites. Although phytohormone metabolites were less abundant than other classes of metabolites, such as flavonoids, amino acids, and sugars and their derivatives, they may play a pivotal role in fruit development and peel coloration in wax apple.

In summary, this study provides a scientific basis for the application of plant growth regulators to improve the quality, flavor, and color of wax apple fruit, thereby enhancing fruit quality and economic value in wax apple cultivation.

## 4. Materials and Methods

### 4.1. Plant Material

The healthy wax apple plants for the experiment were approximately 8 years old, named “Taiguozihong”, and were cultivated in a greenhouse under a root-limited culture model at Fujian Institute of Subtropical Botany, Xiamen, Fujian, China. The plants were treated by drought stress and ring cutting in mid-November. After treating for 45 days, they were sprayed with flowering promoter and also sprayed with potassium and phosphate twice a week to provide nutrients and promote flowering. Flower buds began to sprout from branch tips and main stems in mid-February of the following year. The fruits and peels were selected as materials at 15 (S1), 30 (S2), 45 (S3), and 60 (S4) days after flower (DAF) (Figure 1). The biological characters and peel color of the fruits were determined. At each fruit development stage, four bunches of fruits (four fruits per bunch) with uniform size and color were harvested from every tree, totaling nine trees. Twenty healthy fruits were selected, and their biological characters and peel color were determined. The peels were sliced with a blade to a thickness of 0.2–0.5 mm and cut into 2.0 × 2.0 mm pieces. The pieces of twenty fruits were mixed, and approximately 0.50 g was placed in a cryogenic tube, frozen in liquid nitrogen, and stored at −80 °C. Each of the samples included three biological replicates.

### 4.2. Biological Character and Peel Color Determination

The transverse and longitudinal diameter of the fruits were measured by vernier caliper (0.01 mm). The weight of a single fruit was determined by an electronic balance (0.01 g), while the volume of a single fruit was measured by the drainage method. Lightness, redness, and yellowness of peels were measured by a color difference meter (CR-400).

### 4.3. Metabolite Analysis by HPLC–MS/MS

Metabolomics analysis was performed by Genedenovo Biotechnology Co., Ltd. (Guangzhou, China), following a previously reported method [24,41]. The procedure began with grinding the samples using a mixer mill and zirconia at 30 Hz for 1.5 min. Subsequently, the ground samples (0.1 g) were extracted with 1 mL 70% methanol solution. The extraction condition was conducted at 4 °C for 12 h. In addition, 0.1 mg·g^−1^ of lidocaine (Sigma-Aldrich, Shanghai, China) was added as an internal standard. The mixture was centrifuged for 10 min at 13,400× *g*-force at 4 °C. The supernatant was collected, filtered through a 0.22 μm membrane and subjected to liquid chromatography tandem mass spectrometry analysis (LC–MS/MS). The LC–MS/MS analysis was conducted using the ExionLCTM AD system (SCIEX) and QTRAP^®^ 6500+ mass spectrometer (SCIEX) (AB Sciex, Framingham, MA, USA; Genedenovo, Guangzhou, China). Chromatographic separation was achieved by injecting the sample into an Xselect HSS T3 column (2.1 mm × 150 mm, 2.5 μm; Waters, Milford, MA, USA) and a 20 min linear gradient was used for both positive and negative polarity modes, with a flow rate of 0.4 mL·min^−1^. The eluent consisted of A and B (A: 0.1% formic acid in water; B: 0.1% formic acid in acetonitrile, Thermo Fisher Scientific, Waltham, MA, USA). The solvent gradient parameters were set as follows: 2% B for 2 min; a gradient from 2% to 100% B over 15 min; 100% B for 17 min; a decrease from 100% to 2% B in 0.1 min; and maintaining at 2% B for 20 min. The mass spectrometry conditions were adjusted to operate in both positive and negative polarity modes. For positive polarity, the curtain gas was set at 35 psi, the collision gas was medium, the ion injection voltage was 5500 V, and the temperature was 550 °C, with the ion source gas ratios of 1:60 and 2:60. Similarly, for negative polarity, the settings were a curtain gas of 35 psi, a collision gas medium, an ion injection voltage of −4500 V, and a temperature of 550 °C, with the same ion source gas ratios.

The method of combining the VIP values of the Fold Change (FC) and the OPLS-DA model was adopted, and the differential metabolites were effectively screened with VIP ≥ 1 as the screening conditions. Cluster analysis and KEGG enrichment analysis were conducted using TBtools v2.326 and the R package v3.23 ClusterProfiler, thereby precisely identifying the key pathways closely related to the expression of the differentially expressed metabolites.

### 4.4. Transcriptomics Analysis

Total RNA was extracted from peels using RNA Plant Plus (Tiangen, Beijing, China) for transcriptome sequencing analysis. RNA integrity and DNA contamination were assessed using a Biometra gel Electrophoresis Apparatus (Göttingen, Germany), and RNA integrity was measured using a Nanophotometer Spectrophotometer (IMPLEN, Westlake Village, CA, USA) and accurately assessed using an Agilent 2100 Bioanalyser (Agilent Technologies, Inc., Santa Clara, CA, USA). RNA concentration was precisely determined using a Qubit 2.0 Fluorometer (Life Technologies, USA). The cDNA library was sequenced by Genedenovo Biotechnology Co., Ltd. on an Illumina Sequencing Platform [41]. The differential expression of genes was analyzed by DESeq2. Our imported data including read counts obtained from gene expression level analysis and the genes that met the selection criterion of *p* < 0.05 and |log_2_ FC| > 1 were regarded as significantly differential expressed genes (DEGs). Venn analysis of DEGs was conducted using the online website. To gain further insights into the phenotypic changes, Phyper (a function of R) was employed to perform Kyoto Encyclopedia of Genes and Genomes (KEGG) enrichment analysis on the annotated DEGs based on hypergeometric test. All the DEGs were submitted to KEGG pathway enrichment analysis for functional annotation, and the genes with Bonferroni Q-value < 0.05 were regarded as significant. Using the online analysis platform OmicShare (www.omicshare.com), we screened out the genes related to the biosynthesis and the signaling of plant hormone signal transduction and showed expression differences and changes in the top 20 significant KEGG pathway to draw a scatter plot for result presentation.

### 4.5. Phytohormones Content Determination

The peels were freeze-dried in a vacuum freeze-dryer (Scientz-100 F) and ground into fine powders using a Retsch MM 400 apparatus. The sample extracts were analyzed by using an LC–ESI–MS/MS system (HPLC: Agilent 1290; MS, AB 6500 Triple Quadrupole, Framingham, MA, USA). The analytical conditions were as follows for LC: column, Agilent Poroshell 120 SB-C18 (150 mm × 2.1 mm i.d., 2.7 μm); solvent system, methanol with 0.1% formic acid (A), water with 0.1% formic acid (B), gradient program, started at 20% A (0 min–1 min), increased to 90% A (1 min–9 min), 90% A (9 min–10.5 min), finally ramped back to 20% A (10.5 min–10.6 min), 20% A (10.6 min–13.5 min); flow rate, 0.2 mL/min; temperature, 40 °C; injection volume 2 μL. The ESI source operation parameters were as follows: ion source, turbo spray; source temperature 400 °C; ion spray voltage (IS) 4.5 kV; curtain gas was set at 15.0 psi; Gas1 was set at 65.0 psi; Gas2 was set at 70.0 psi; monitor model—MRM. The internal standard method was used to determine hormone contents. Analyses of phytohormones were assayed by Convinced-Test Company (www.convinced-test.com). Three biological repeats were made for each sample.

### 4.6. Quantitative Real-Time PCR Analysis

Total mRNA was extracted from the frozen samples collected using TRIzol Reagent (Invitrogen, Thermo Fisher Scientific, Waltham, MA, USA), and cDNA synthesis was performed using PrimeScript^®^ RTReagent kit (TaKaRa, Shiga, Japan). All primers involved in the qRT–PCR process were designed using the online tool Primer 5.0 Plus, and the specificity of the primer was examined by the corresponding dissociation curves and gel electrophoresis (Supplementary Table S4). Transcript levels were quantified using a CFX96 Real-Time PCR Detection System (Bio-Rad, Hercules, CA, USA) with the intercalating dye SYBR-green. Different stage peel samples were replicated three times. The 2−ΔΔct technique was utilized to calculate the gene expression level, with EF-1α serving as the reference gene. 

### 4.7. WGCNA Analysis and Gene Network Visualization

Weighted gene coexpression network analysis (WGCNA facilitated the construction of a coexpression network [42], which is an analysis method for the gene expression patterns of multiple samples. It can cluster genes with similar expression patterns and analyze the correlation between each module and the specific traits or phenotypes. Coexpression networks were constructed using the WGCNA (v1.47) package in R. After filtering the genes with low expression, gene expression values were imported into WGCNA to construct coexpression modules. To ensure compliance with the scale-free network distribution, an appropriate weight coefficient β value was calculated based on the pick SoftThreshold function in the WGCNA package. It was determined that when the soft threshold (β) was 8, the gene expression network conforms to the scale-free topological network. The clustering tree of the gene system was drawn based on topological overlap TOM to present the clustering relationship of each gene. The gene coexpression modules were divided using the dynamic hybrid cutting algorithm, with the parameters set as min Module Size = 30 and merge Cut Height = 0.25. Different colors represent different modules. Gray indicates genes that are not classified into a module, and this module is meaningless. The vertical distance represents the distance between two nodes (genes), while the horizontal distance has no practical significance.

### 4.8. Statistical Analysis

Data on phytohormone content, FPKM, and qRT–PCR were analyzed using one-way analysis of variance (ANOVA) with SPSS 19.0 software. Statistically significant differences were assessed using the least significant difference test, with the significance set at *p* < 0.05. Bioinformatics analysis and mapping were executed using OmicShare tools, while a network diagram visualizing the relationships between genes and metabolites was created using using the WGCNA (v1.47).

## Figures and Tables

**Table 1:** Phytohormone-related metabolites in peels of wax apple at different stages.

Kinds	Compounds	log2 FC		
		S2-vs-S1	S3-vs-S2	S4-vs-S3
IAA	indoleacetic acid–glutamic acid	−0.17	1.03	−0.24
CTKs	9-β-glucosyl-N6 -(isoprene) adenine	−0.17	0.28	0.90
	isopentenyl adenosine	−1.94	0.66	0.26
	isopentenyl-7-glucoside	0.04	−0.18	1.13
GAs	gibberellin A7	−0.14	1.40	0.05
	gibberellin A9	−0.90	−0.30	0.08
	gibberellin A20	−1.31	0.84	−1.10
JAs	jasmonic acid	−0.36	−0.70	−0.16
	12-hydroxyjasmine monoacyl-L-isoleucine	−0.27	−0.35	−0.21
	(-)-jasmonoyl-L-isoleucine	−0.04	−1.74	−0.10
	N-[(-)-jasmonoyl]-(L)-isoleucine	−0.59	−0.51	0.23
ZRs	trans-zeatin 9-O-glucoside	−0.06	0.68	0.69
	trans-Zeatin riboside	−0.20	−0.14	−0.39
	trans-zeatin N-glucoside	−0.48	0.69	−0.11
ABA	abscisic acid	0.21	−1.95	−0.15
ACC	1-aminocyclopropane carboxylic acid	−0.19	0.56	0.06
SA	salicylic acid O-glucoside	−0.45	0.77	−0.12

## Data Availability

RNA-seq data that support the findings of this study were deposited in the National Biotechnology Center Archives database with the primary accession code PRJNA1313141. (http://www.ncbi.nlm.nih.gov/bioproject/PRJNA1313141, accessed on 23 October 2026). Further inquiries can be directed to the corresponding authors.

## Supplementary Materials

The following supporting information can be downloaded at: https://www.mdpi.com/article/10.3390/plants15213394/s1, Supplementary Table S1. Base quality statistics of transcriptome datas in peel of wax apple; Supplementary Table S2. Differentially expressed genes of peels in different comparison; Supplementary Table S3. WGCNA Analysis of phytohormone-related genes and metabolites; Supplementary Table S4. Primers used in this study.
